# Peer-reviewed visuals and auditory – isn’t it time to incorporate?

**DOI:** 10.1080/0886022X.2023.2274507

**Published:** 2023-10-26

**Authors:** Karim Soliman, Charat Thongprayoon, Wisit Cheungpasitporn

**Affiliations:** a Division of Nephrology, Department of Medicine, Medical University of South Carolina, Charleston, SC, USA; b Medical Services, Ralph H. Johnson VA Medical Center, Charleston, SC, USA; c Division of Nephrology and Hypertension, Department of Medicine, Mayo Clinic, MN, USA

**Keywords:** Education, visuals, videos, auditory, artificial intelligence

## Abstract

A remarkable opportunity emerges amidst the dynamic evolution of medical education, one that could fundamentally alter how healthcare professionals gain and share knowledge. The concept of incorporating a structured, peer-reviewed video and audio section, as well as a dedicated submission portal, into the medical journals symbolizes a revolutionary advance. This addition has the potential to not only improve the educational experiences of the journal’s audience, but also to create a more accessible forum for the exchange of knowledge and citation. In this article, we explore the compelling potential of introducing structured videos and podcasts into the domain of medical literature, as well as the promising implications for revamping medical practitioners’ learning strategies.

## Introduction

In light of the swiftly changing landscape of medical education, there is a remarkable opportunity to transform how healthcare professionals acquire and disseminate knowledge. The incorporation of a structured, peer-reviewed video and auditory section with a submission portal into medical journals could represent a significant leap forward. This addition has the potential to not only improve the educational experiences of the journal’s followers, but also facilitate a more accessible platform for knowledge sharing and citation. This perspective investigates the promising implications of incorporating structured videos and podcasts contents into medical literature and its potential to revolutionize medical practitioners’ learning strategies.

## Discussion

In recent years, the medical education landscape witnessed a profound transformation. Traditional methods of disseminating knowledge through the reading of manuscripts and listening to podcasts are progressively being replaced by formats that are more interactive and engaging. This shift is attributable to the impact of social media, the rise of artificial intelligence, and the growing preference for visual and animated educational materials.

**Figure 1.**
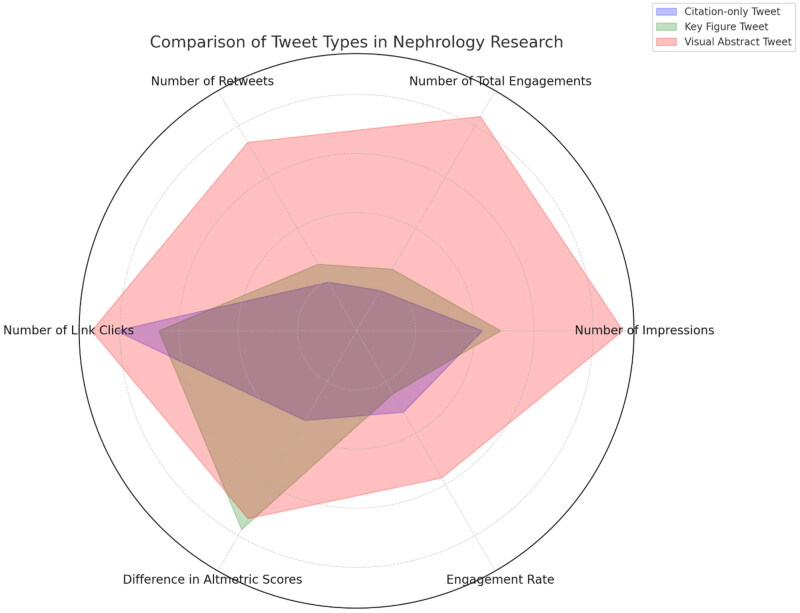
Superiority of visual abstracts across most metrics than citation-only tweets.

Numerous studies and articles have examined the effect of these changes on medical education and the amount of time physicians devote to social media platforms and visual content [1]. Latif et al. highlighted how medical professionals are increasingly using platforms such as Twitter to share medical knowledge, whereas Curran et al. discussed the rising popularity of medical vlogs on YouTube, emphasizing their lack of peer-review [2,3]. YouTube now verifies channels of certified healthcare professionals based in the US, UK and other areas for added credibility and trustworthiness [4]. Hurtubise et al.’s study also pointed out the benefits of employing video-based learning in medical education, such as enhanced comprehension and memory of challenging medical ideas [5]. The research conducted by Oska et al. [6] provides empirical evidence that significantly enriches our perspective on how medical information can be effectively disseminated. Their work conclusively shows that visual abstracts significantly increase the visibility and interaction rates of nephrology research on Twitter (Figure 1). This research aligns well with the growing trend of using various forms of visual media—from YouTube medical content to AI-customized learning resources—for medical communication and education. Given the strong evidence of the impact of visual abstracts, both medical professionals and scholarly journals would do well to incorporate them more frequently into their strategies for disseminating information. Similarly, Seo et al. emphasized the role of artificial intelligence in tailoring educational content to individual students, thereby increasing its effectiveness and engagement [7]. Further, the video section could be subcategorized into challenging cases, comparing most recent studies discussing a particular topic, and concise educational materials for patients.

**Figure 2.**
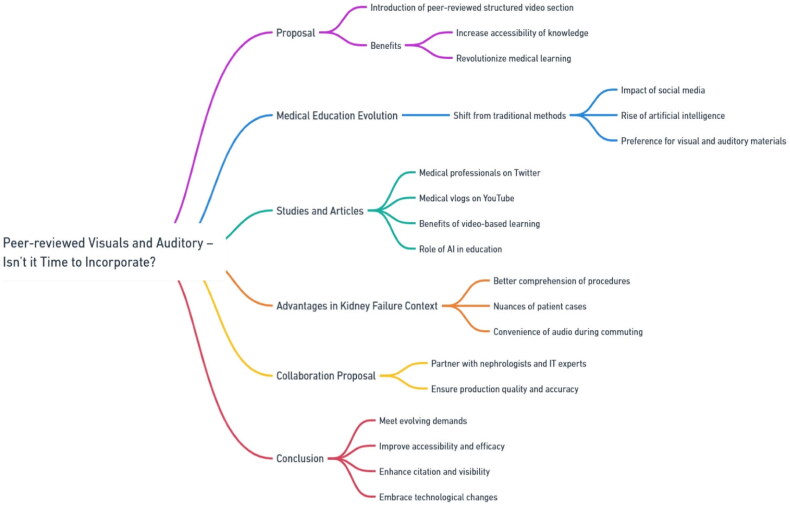
Illustrative mind map for peer-reviewed visuals and auditory.

Videos can be particularly advantageous to both patients and medical professionals in the context of kidney disease [8]. Through visual and auditory demonstrations, procedures such as hemodialysis, peritoneal dialysis, kidney biopsy, and kidney transplantation can be better comprehended. Moreover, video content can vividly portray the complexities and decision-making challenges that clinicians face in actual patient cases. Incorporating peer-reviewed videos and podcasts into educational resources is crucial. Not to mention the convenience and ease of listening to the audio, during traffic and commuting where reading would be impractical. Figure 2 illustrates a mind map for peer-reviewed visuals and auditory.

We propose collaborating with professional nephrologists and informational technologists to help build a peer-reviewed structured video category. This collaboration would ensure the video and auditory content’s production quality, accuracy, and validity, aligning it with the journal’s high standards.

In conclusion, the incorporation of a peer-reviewed structured video and podcast section to medical journals would not only meet the evolving demands of medical professionals and patients, but it would also improve the accessibility and efficacy of medical education. This will also significantly enhance citation and visibility rates, as well as attract a broader readership. It is critical that we embrace these technological changes in order to continue offering high-quality education to our patients and healthcare providers as we evolve in the digital age.
